# Comparative Cytology of Female Meiosis I Among *Drosophila* Species

**DOI:** 10.1534/g3.120.400867

**Published:** 2020-03-26

**Authors:** Ahmed Majekodunmi, Amelia O. Bowen, William D. Gilliland

**Affiliations:** Department of Biological Sciences, DePaul University, 2325 N. Clifton Ave., Chicago IL 60614

**Keywords:** Chromosomes, Meiosis, Drosophila, Heterochromatin, Comparative Biology

## Abstract

The physical connections established by recombination are normally sufficient to ensure proper chromosome segregation during female Meiosis I. However, nonexchange chromosomes (such as the Muller *F* element or “dot” chromosome in *D. melanogaster)* can still segregate accurately because they remain connected by heterochromatic tethers. A recent study examined female meiosis in the closely related species *D. melanogaster* and *D. simulans*, and found a nearly twofold difference in the mean distance the obligately nonexchange dot chromosomes were separated during Prometaphase. That study proposed two speculative hypotheses for this difference, the first being the amount of heterochromatin in each species, and the second being the species’ differing tolerance for common inversions in natural populations. We tested these hypotheses by examining female meiosis in 12 additional *Drosophila* species. While neither hypothesis had significant support, we did see 10-fold variation in dot chromosome sizes, and fivefold variation in the frequency of chromosomes out on the spindle, which were both significantly correlated with chromosome separation distances. In addition to demonstrating that heterochromatin abundance changes chromosome behavior, this implies that the duration of Prometaphase chromosome movements must be proportional to the size of the *F* element in these species. Additionally, we examined *D. willistoni*, a species that lacks a free dot chromosome. We observed that chromosomes still moved out on the meiotic spindle, and the *F* element was always positioned closest to the spindle poles. This result is consistent with models where one role of the dot chromosomes is to help organize the meiotic spindle.

In addition to the use of *D. melanogaster* as a model organism for genetic and developmental studies, the *Drosophila* genus is estimated to contain several thousand species, a number of which have had their genomes sequenced (Drosophila 12 Genomes Consortium *et al.* 2007; Miller *et al.* 2018), making it an important model for comparative and evolutionary studies. The ancestral condition is six pairs of telocentric chromosomes, while the derived genomes of extant species typically have between 4 and 6 pairs of homologous chromosomes (Ashburner *et al.* 2005). For example, the derived condition in *D. melanogaster* is one pair of sex chromosomes plus three pairs of autosomes, two of which are large metacentric chromosomes while the third is a small “dot” chromosome. Some of the first comparative genomics studies ever performed were done in *Drosophila*, when linkage maps of morphological traits were used to demonstrate homology among the chromosome arms between pairs of closely-related *Drosophila* species (Donald 1936; Sturtevant and Tan 1937), which were combined with the discovery that banded salivary gland chromosomes could be used to identify and compare genomic regions more accurately (Tan 1935). However, as chromosome numbers are assigned from largest to smallest within each species, early comparative analyses were hampered by variation in the nomenclature. This problem was addressed by H.J. Muller who labeled each *D. melanogaster* chromosome arm as a lettered element (chromosome arm equals Muller element: *X = A*, *2L = B*, *2R = C*, *3L = D*, *3R = E*, *4 = F*) and then used the conserved salivary gland banding patterns to locate those elements in other species of flies (Muller 1940). While this revealed that there had been multiple rearrangements and changes in chromosome number among these species, the six Muller elements were largely conserved intact and element *F* was found to remain as a small “dot” chromosome in most species. Subsequent genomic analysis has shown that while inversions can rearrange the order of genes within the Muller elements, ∼95% of genes are consistently located within the same element across species (Bhutkar *et al.* 2008).

One function that chromosomes must be able to carry out is to segregate through meiosis, a specialized form of the eukaryotic cell division cycle. During meiosis, diploid cells replicate their chromosomes once then divide twice, producing four haploid sets of chromosomes. The first meiotic division is unique, as homologous chromosomes must segregate from each other, whereas it is sister chromatids that separate in both Mitosis and Meiosis II. While in males all four products of meiosis become functional sperm, in females only one of the four becomes the egg pronucleus, with the other three forming the polar bodies. It has been hypothesized that selfish genetic elements could orient themselves to increase their odds of becoming the pronucleus (at the cost of a higher rate of segregation errors that is deleterious to the host), providing an opportunity for meiotic drive to induce an evolutionary arms race between selfish genetic elements and their suppressors (Rice 2013; Ross and Malik 2014).

The failure to accurately segregate chromosomes results in aneuploidy, which is the most frequent cause of human birth defects, resulting in Down syndrome, Turner syndrome, and many miscarriages (Hassold and Hunt 2001). The process of homologous recombination (also called crossing over or exchange) physically locks homologous chromosomes together, and is usually necessary and sufficient for accurate segregation (Zhang and Hawley 1990). However, nonexchange chromosomes can still segregate accurately thanks to the distributive segregation system, which has been most extensively studied in *D. melanogaster* (Hughes *et al.* 2018). During Prometaphase I, nonexchange chromosomes move out onto opposite sides of the spindle; while it was originally thought that Metaphase arrest was reached with the chromosomes out (Theurkauf and Hawley 1992), it was later shown that the nonexchange chromosomes actually undergo congression prior to Metaphase arrest, moving in to join the exchange chromosomes at the Metaphase plate (Gilliland *et al.* 2009). To establish proper co-orientation with their homologs, nonexchange chromosomes appear to remain connected by heterochromatin threads acting as tethers (Hughes *et al.* 2009; Hughes and Hawley 2014). These tethers have also been observed connecting chromosomes undergoing secondary nondisjunction (*XX*⇔*Y* segregation in *XXY* females) as well as heterologous segregations (*e.g.*, *C(1)RM*⇔*C(4)RM* (Gilliland, Colwell, Lane, *et al.* 2015) or *C(1)RM*⇔*C(2)EN* (Gilliland *et al.* 2016)).

In *D. melanogaster* the dot chromosome never undergoes recombination, with experimental samples in excess of one million progeny showing zero cases of homologous crossovers (McMahan *et al.* 2013). This causes the dot chromosome to be frequently located out on the spindle during Prometaphase. While crossing over can occur on the *F* element in *D. melanogaster* females that are triploid (Sturtevant 1951) or mutant for BLM helicase (Hatkevich *et al.* 2017), other species (including *D. virilis* and *D. erecta*) have been observed to undergo low levels of *F* element meiotic recombination under normal circumstances (Leung *et al.* 2015). The mechanism of this suppression of recombination on the *F* is not well understood; while experiments with translocations or species with natural *F* element fusions show that this suppression of recombination can spread along a chromosome depending on genomic context (Powell *et al.* 2010), a number of exceptions (such as the greatly expanded *F* element in *D. ananassae)* demonstrate that neither chromosome size nor the centromere effect can be the sole explanations for how recombination is suppressed on this chromosome (Riddle and Elgin 2018).

A recent study published in genetics (Gilliland, Colwell, Osiecki, *et al.* 2015) examined the dot chromosome during female meiosis in *D. melanogaster* and its close relative *D. simulans*, as well as interspecies hybrids where the *D. simulans* dot chromosome had been introgressed into an otherwise *D. melanogaster* genetic background. While males homozygous for the foreign chromosome were sterile, homozygous females were fertile, and the introgressed chromosome still segregated accurately, both when homozygous as well as when heterozygous with a *D. melanogaster* chromosome *4*. In images of fixed Prometaphase oocytes, that study also observed a nearly twofold reduction in the mean separation distance between the dot chromosomes, both in pure-strain *D. simulans* and in *D. melanogaster* females carrying the introgressed chromosome. The authors proposed two speculative hypotheses about differences between these species that might explain this observation. First, *D. melanogaster* has a greater amount of total heterochromatin throughout its genome than *D. simulans* (Ferree and Barbash 2009). As the tethers connecting nonexchange chromosomes contain heterochromatin (Hughes *et al.* 2009), then perhaps having more heterochromatin would permit longer tethers, analogously to being able to pull the two ends of a rope farther apart when there is more rope. Consistent with this, Gilliland *et al.* also observed that *D. melanogaster* females heterozygous for *Df(4)m101-62f*, a deletion that removes much of the pericentric heterochromatin from chromosome *4*, also had much shorter dot-dot separation distances. The second interspecies difference that was proposed is that while *D. melanogaster* natural populations harbor common cosmopolitan inversions at an average of ∼1 inversion per fly, *D. simulans* populations lack cosmopolitan inversions and carry only ∼1 inversion per 200 flies (Lemeunier and Aulard 1992). As inversions block crossing over, increasing the abundance of inversions would make meiosis with nonexchange chromosomes more common, which would result in nonexchange chromosomes moving out on the spindle more frequently. As the dot chromosomes are positioned closer to the spindle poles than other nonexchange chromosomes in *D. melanogaster* (Gilliland, Colwell, Lane, *et al.* 2015), having a greater dot-dot separation may provide more space for nonexchange chromosomes to establish proper coorientation, and could be part of the mechanism that allows *D. melanogaster* populations to tolerate inversions much better than *D. simulans* populations.

Please note that while very little is known about what causes species to be monomorphic or polymorphic for inversions, there are several reasons to assume that this is due to one or more fixed differences between species, and not a property of the inversion chromosomes themselves. First, while multiply inverted balancer chromosomes that prevent recombination have been a critical tool for research in *D. melanogaster* for decades (Ashburner *et al.* 2005), the construction of balancers that block recombination in *D. simulans* has proved impossible. Only one *D. simulans* balancer chromosome has ever been reported, a chromosome bearing the single inversion *In(3R)Ubx* (Coyne and Sniegowski 1994), which upon subsequent testing was found to actually not act like a balancer, as recombination outside the inversion was only mildly decreased when heterozygous with a normal sequence chromosome (Jones and Orr 1998). Whereas inversion chromosomes in *D. melanogaster* were first discovered by their suppressive effect on recombination rates (Sturtevant 1919; Ashburner *et al.* 2005), no other strains of *D. simulans* have been found that can act like balancers. This suggests that the ability for inversions to suppress recombination is probably a difference in the meiotic machinery that is fixed between these species. Second, a study of mammalian karyotypes that categorized the proportion of acrocentric chromosomes found a strongly U-shaped distribution, where most species were close to either 0% or 100% acrocentric chromosomes (de Villena and Sapienza 2001). This result was in stark contrast to their expectation of a bell-shaped curve centered on 50%, which would occur if each chromosome could transition randomly and independently between being acrocentric or bi-armed (metacentric or submetacentric). The authors of that study concluded that meiotic drive was the best explanation for the observed pattern of karyotype differences, with different ‘spindle polarities’ that favored the transmission of either acrocentric or bi-armed chromosomes, and that populations could change spindle polarity on very short (< 500 year) time scales, presumably as a result of an allele sweeping through the population due to meiotic drive. Combining these examples with the phylogeny of the small number of species in this study (which clearly shows that species must frequently transition between being polymorphic or monomorphic for inversions), our working model is that the presence or absence of inversions is most likely to be caused by a similar type of unknown fixed difference in the meiotic machinery between species, with species changing between states on very short time scales (as flies have much shorter generation times than mammals, the transitions should occur much faster as well) as new variants sweep through populations, either due to selection or meiotic drive. Like with spindle polarity favoring or disfavoring the transmission of acrocentric chromosomes, if this difference in inversion tolerance has an effect on the dot-dot separation distance on the meiotic spindle, then our examination of a single strain from each species ought to be sufficient to test this hypothesis.

While the two hypotheses were admittedly speculative, we set out to test them, reasoning that even if neither hypothesis were supported, we would still be characterizing female meiosis in many of these species for the first time. We sampled an additional 12 *Drosophila* species, with and without common inversions, and measured their average dot-dot chromosome distances during female meiotic Prometaphase I, to see if those distances correlated with either the abundance of inversions, the amount of genomic heterochromatin, or both. We found very little support for either of these initial hypotheses; there was no significant correlation between the dot-dot separation distances and either the amount of genomic heterochromatin or with the abundance of common inversions among these species. However, while characterizing meiosis in these species, we observed significant variation in the apparent size of the dot chromosomes, with the largest species *(D. similis)* having a cross-sectional area over 10 times greater than that of the smallest *(D. hydei)*. We also observed over fivefold differences between species in the proportion of oocytes that had chromosomes positioned out on the spindle. These measurements were both correlated with dot-dot distances, but also strongly correlated with each other. This demonstrates that larger dot chromosomes tend to separate farther from each other during Prometaphase. Additionally, because these were fixed samples, the percentage out result implies that species with larger dot chromosomes spend a greater amount of time undergoing the Prometaphase chromosome movements than species with smaller dots.

Finally, we also examined female meiosis in *D. willistoni*, a species where the *F* element has fused with element *E* (the equivalent of *3R* in *D. melanogaster)* to form chromosome *3* in that species (Schaeffer *et al.* 2008). Despite lacking a free dot chromosome, and undergoing recombination on the third chromosome (Powell *et al.* 2010), we nevertheless still saw nonexchange chromosomes positioned out on the spindle in ∼12% of oocytes, and in each figure, the *F* elements were positioned closest to the spindle poles. This observation is consistent with a model where one of the functions of the dot chromosomes is to help organize the Prometaphase chromosome movements along the meiotic spindle (Gilliland, Colwell, Lane, *et al.* 2015).

## Methods

### Species selection

We selected species that had been categorized as either monomorphic or polymorphic for inversions (Sperlich and Pfriem 1986), based on whether the same inversions were recovered multiple times. Note that this includes inversions located anywhere in the genome, and not just on the *F* element. We also identified species where the percentage of heterochromatin in the genome was measured, based on the percentage of repeat sequences in their whole genome sequencing data (Bosco *et al.* 2007). Two species, *D. hydei* and *D. americana*, had not been characterized by whole genome sequencing, so the percentage of underreplicated sequences as measured by DAPI was used instead for those species (ibid.). Note that this measurement is for all heterochromatin throughout the genome, and assumes that the amount located on the *F* element should be proportional to the genomic average. To control for phylogenetic similarity, we preferred species pairs that, like *D. melanogaster* and *D. simulans*, were closely related but differed in the presence of common inversions in their natural populations. Because more published data were available for inversions than heterochromatin content, we selected some species that did not yet have their heterochromatin characterized, reasoning that they could be characterized later if the pattern looked promising. The *D. melanogaster* and *D. simulans* stocks had been maintained in our lab since the previous study (Gilliland, Colwell, Osiecki, *et al.* 2015) while stocks of the new species were obtained from the Drosophila Species Stock Center at UC San Diego (now located at Cornell University), except for *D. virilis* which was a gift from Dr. Justin Blumenstiel (KU Lawrence) (see Table S1 for strain identifiers). All stocks were raised on the growth media recommended by the Stock Center. Food recipes used were Bloomington Formula (premade powder mix from Genesee Scientific prepared per manufacturer’s instructions, with propionic acid and Tegosept added as antifungal agents), as well as Banana Food and Banana-Opuntia Food, which were prepared according to the Species Stock Center recipes (Table S1). The phylogeny of these species with divergence times was built using the TimeTree database on 7/29/2019 (Kumar *et al.* 2017); all species were present in their database except for *D. similis*. As this species is a member of the *dunni* subgroup (Cordeiro *et al.* 2014), *D. dunni* was used to construct the tree, as this species would have the same position and divergence times relative to the other species as *D. similis*. The TimeTree image was then edited in Adobe Illustrator to replace the *D. dunni* label with *D. similis*, color code species for their inversion types, and reorder the clades by flipping the basal-most node.

### Ovary preps

Bottles of each stock were cleared of adults, and newly-eclosed adults were collected 6 hr later. To dissect females of each species at similar time points in development, when many oocytes would be in Prometaphase, we dissected females shortly before they began laying fertilized eggs. In *D. melanogaster*, mated females had the highest proportion of Prometaphase oocytes at 2 days post eclosion (dpe) (Gilliland *et al.* 2009). For most species, females were 42-48 hr post eclosion at the point of dissection; the exceptions (*D. meridiana*, *D. americana* and *D. nigricruria)* were dissected at 3.5 dpe. To standardize prep conditions, a timer was started as the flies were anesthetized with CO_2_, followed by hand-dissection of ovaries as quickly as possible in room temperature 1x Robb’s media + 1% BSA (Sullivan *et al.* 2000), transferring ovaries to a second well of media after extraction. For each prep, 10 females were dissected, incubated in Robb’s until 7 min. from the start, then buffer and ovaries were transferred to a 1.5 ml Eppendorf tube and allowed to settle. At 8 min., the Robb’s was aspirated and 1.3 mL of room temperature fixative, a 1:1 mixture of 16% EM grade paraformaldehyde (Ted Pella) plus William’s Hypotonic Oocyte Preservation and Stabilization Solution (Gillies *et al.* 2013) combined immediately before use, was applied. After shaking on a nutating mixer for 5 min, oocytes were allowed to settle for 1 min., fixative was aspirated off, oocytes were washed briefly in PBST (PBS + 0.1% Triton-X 100), and ovarioles were separated by rapidly pipetting up and down with a p1000 pipette. Oocytes were then washed 3x in PBST for 15 min. each, stained in PBST plus DAPI for 6 min., washed again in PBST (3x quickly followed by 2x for 15 min.) then mounted on slides in SlowFade Gold (Invitrogen) with the coverslip edges sealed with nail polish. Oocytes for immunofluorescent preps were fixed as above, dechorionated by rolling between frosted glass slides, incubated in rat anti-tubulin (Serotec MCA786, 1:250) and rabbit anti-phosphorylated histone H3 at serine 10 (Millipore 06-570, 1:500) primary antibodies, followed by goat anti-rat-IgG conjugated to Alexa 647 (1:250, Invitrogen) and goat anti-rabbit-IgG conjugated to Alexa 568 (1:250, Invitrogen), per previously published protocols (Hughes *et al.* 2009). Note that we have not assayed the pH3S10 antibody specificity, as we are using it as a cytological marker that highlights the faint threads, which does not depend on the actual epitope.

### Mitotic chromosome preparation

Mitotic chromosomes from *D. similis* were prepared from third instar larvae using standard brain squash protocols (Sullivan *et al.* 2000) except that chromosomes were stained by mounting with SlowFade Gold Plus DAPI mounting media (Molecular Probes).

### Imaging and quantification

Oocytes were imaged on a Leica TCS SPE II confocal microscope running Leica LAS software (www.leica.com). To ensure oocytes were not missed or double counted, microscope slides were photographed on a dissecting microscope, and a printout was used as a map to mark oocytes at 10x magnification using the Mark and Find function. Once marked, confocal image stacks of oocyte chromosomes were collected at 63x magnification with a standardized confocal zoom setting to yield 54nm pixel sizes (1.7x the Nyquist resolution limit). Presented images were deconvolved using Huygens Essential (www.svi.nl) with an estimated PSF and all parameters default except mounting media refractive index, which was 1.42 per manufacturer.

Calculation of dot-dot distances was done in Excel by combining XY distances (measured from outer chromosome edges using the LAS line tool on projected image stacks) with Z distances (determined by multiplying the number of confocal sections between the centers of the dot chromosome light cones in orthogonal projections by the section thickness) using the Pythagorean theorem (distance = sqrt(d^2^ + z^2^), where d is the measured XY distance in the projected image). Measurement was restricted to oocytes that had at least one dot chromosome out on the spindle, with the other locatable. Since chromosomes do not have completely sharp edges, a chromosome was classified as “out” if there was at least a 50% dip in background-subtracted fluorescent intensity between the dot and the space between the dot and the adjacent chromosome, using the LAS Line ROI profile tool. Oocytes with both dot chromosomes on the same side of the spindle, with additional nonexchange chromosomes, or other abnormal configurations such as autosomal slippage (Hughes *et al.* 2011) were not used in distance measurements, as those dot-dot distances could be affected by the arrangement of the other chromosomes, but were included in the proportion of cells with chromosomes out. Our target was to obtain measurements from at least 30 oocytes with at least one chromosome out on the spindle for each species, but once that target was reached all remaining oocytes marked on the slide map were still scored. As *D. willistoni* does not have a free dot chromosome, we scored all oocytes from 15 females dissected at 2 dpe, to estimate the proportion of oocytes with any chromosomes out.

To calculate dot chromosome sizes, projected Z-stacks of figures with dot chromosomes out on the spindle were measured using the LAS freehand ROI tool, to estimate the pixel area of at least 20 chromosomes for each species with free dot chromosomes.

To calculate the proportion of oocytes with chromosomes out on the spindle, the total number of oocytes with chromosomes out was divided by the total number of oocytes that had been marked and identified as having matured past Prophase, based on the growth of the oocyte dorsal appendages (Gilliland *et al.* 2009).

### Data analysis

Statistical calculations and plots were done in R (http://cran.r-project.org). Multiple testing correction in Table 2 was performed using the False Discovery Rate procedure (Benjamini and Hochberg 1995) which sets the cutoff for significance at the largest *p* value where *p_i_ < (i/m)*Q, where *i* is the ordered rank of the *p* value, *m* is the number of tests and *Q* is set to *1/2m*.

### Data availability

All numerical measurement data, and the script files used to create figures, are included as supplemental material via FigShare (Supplemental File 2). Supplemental material available at figshare: https://doi.org/10.25387/g3.11862432

## Results

**Figure 1:**
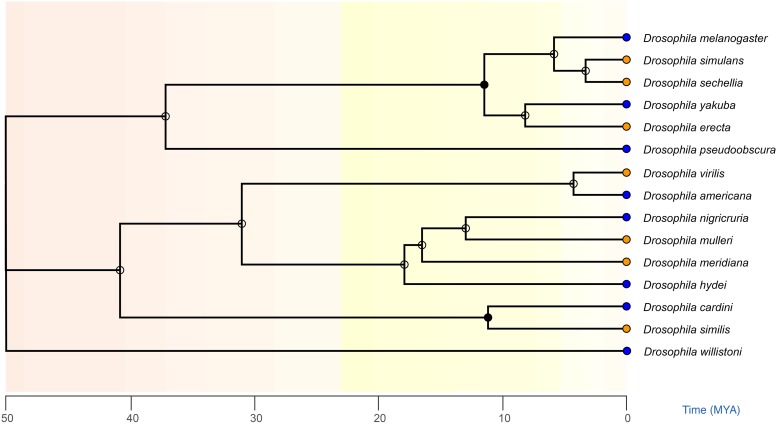
Phylogeny of species used in this study. The TIMETREE database (Kumar *et al.* 2017) was used to create a phylogeny showing the relatedness of all species used in this project. Blue dots indicate polymorphic species that harbor common inversions (like *D. melanogaster)* while orange dots indicate monomorphic species without common inversions (like *D. simulans)*. All species except *D. similis* were in the TIMETREE database; another species in the database that would have had the same relative position in this phylogeny *(D. dunni)* was used to construct the tree.

We identified twelve additional species with free dot chromosomes, six monomorphic species that lacked common chromosomal inversions in natural populations plus six polymorphic species that harbor common inversions (Sperlich and Pfriem 1986), with divergence times ranging from 3 to 50 million years (Figure 1), as well as estimates of the percentage of the genome that was heterochromatic for 9 of them (Bosco *et al.* 2007). We then imaged at least 30 oocytes with at least one dot chromosome out on the spindle, and measured the distance between the dot chromosomes. These data are summarized in Table 1.

**Table 1:** A summary of all data for each species used in this study, including the species name and abbreviation, percentage of the genome that is heterochromatin (%HC), whether the species harbors common inversions, the mean dot-dot separation distance (in µm), the number of oocytes sampled, and the mean size of the dot chromosomes (µm). *: Data for this species taken from (Gilliland, Colwell, Osiecki, *et al.* 2015). n.d.: not done, as this species lacks a free dot chromosome

Species Name (abbreviation)	% HC	Common Inversions	Mean dot-dot distance (µm)	Oocytes with 1+ out	Total oocytes sampled	Proportion out on the spindle	Mean dot chromosome size (µm^2^)
*D. virilis (vir)*	44	No	7.17	38	280	0.15	0.29
*D. americana (ame)*	26	Yes	6.47	38	192	0.20	0.30
*D. sechellia (sec)*	24	No	7.81	33	185	0.18	0.39
D. melanogaster* (mel)	24	Yes	11.33	71	147	0.48	0.85
*D. yakuba (yak)*	23	Yes	7.48	31	92	0.34	0.70
*D. hydei (hyd)*	22	Yes	4.74	31	330	0.09	0.15
D. simulans* (sim)	17	No	6.15	35	80	0.44	0.64
*D. pseudoobscura (pse)*	14	Yes	5.67	35	220	0.16	0.34
*D. erecta (ere)*	9	No	5.93	30	269	0.11	0.26
*D. cardini (car)*	—	Yes	8.40	34	120	0.28	0.72
*D. meridiana (mea)*	—	No	5.44	34	209	0.16	0.29
*D. mulleri (mul)*	—	No	5.37	37	160	0.23	0.39
*D. nigricruria (nic)*	—	Yes	5.93	34	240	0.14	0.22
*D. similis (sig)*	—	No	9.83	33	70	0.47	2.01
*D. willistoni (wil)*	12	Yes	n.d.	14	115	0.12	n.d.

**Figure 2:**
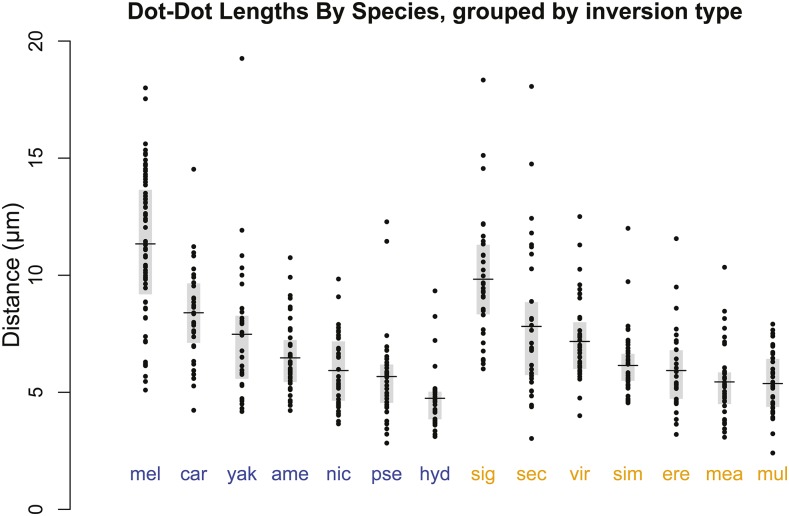
Dot-dot distances by species. For each species (see Table 1 for abbreviations), each dot represents one oocyte measurement, with the mean value (horizontal lines) and inner quartiles (gray boxes) indicated. All figures had at least one dot chromosome fully out on the spindle, with the other locatable. Blue and orange labels represent species with and without common inversions, respectively. While the overall average for all species with common inversions was ∼5% greater than species without (7.15 µm *vs.* 6.82 µm), that difference was not statistically significant (*t*-test, *P* = 0.75).

We first asked whether the presence of common inversions correlated with greater dot-dot distances across *Drosophila* species (Figure 2). Because comparative data from related species are not statistically independent (Felsenstein 1985), we first examined the pairs of closely related species with divergent inversion types; because *D. pseudoobscura* and *D. sechellia* did not have a closely paired species in our sample, this left six comparisons (Table 2). Three comparisons had a greater mean distance in the polymorphic species, while the other three had a greater mean distance in the monomorphic species, a result that fails to support the prediction that species with common inversions would have greater separation distances. Of the pairwise *t*-tests, the *mel-sim* comparison was highly significant (*P* < 10^−15^) while the *yak-ere* and *car-sig* comparisons were both moderately significant (*P* < 0.02); note that for the *car-sig* contrast it was the monomorphic species with a greater mean in the comparison, a result at odds with the predicted direction. If we assume phylogeny can be neglected and just compare the means for all species with and without common inversions, we see that while the average separation distance among the species with common inversions was around 5% greater than the species without, the difference between the two groups was not statistically significant (*t*-test of species means, *P* = 0.75). We also observed a similar amount of variation among the species within each group (Figure 2). Together, these data fail to support the hypothesis that the presence of common inversions is related to the dot-dot separation distance.

**Table 2:** Pairwise Comparison of Inversion-type Divergent Pairs. Pairwise *t* tests were conducted between closely related species pairs, with the prediction that separation distances (using Table 1 values and calculated as *poly – mono*) would be greater in the polymorphic species. However, half the contrasts found a larger separation in the monomorphic species instead. While some of the differences between species pairs were statistically significant (asterisks) even after False Discovery Rate (FDR) adjustment for multiple testing, the overall pattern does not support the hypothesis that inversion tolerance is related to dot-dot distance

Polymorphic Species	Monomorphic Species	Mean ∆, µm	*t* test p value	FDR Cutoff
*D. melanogaster*	*D. simulans*	5.2	*P* < 2.2 x 10^−16^*	0.014
*D. yakuba*	*D. erecta*	1.6	*P* = 0.017*	0.027
*D. cardini*	*D. similis*	−1.4	*P* = 0.018*	0.041
*D. hydei*	*D. meridiana*	−0.70	*P* = 0.065	0.056
*D. americana*	*D. virilis*	−0.70	*P* = 0.0755	0.069
*D. nigricruria*	*D. mulleri*	0.56	*P* = 0.1145	0.083

**Figure 3:**
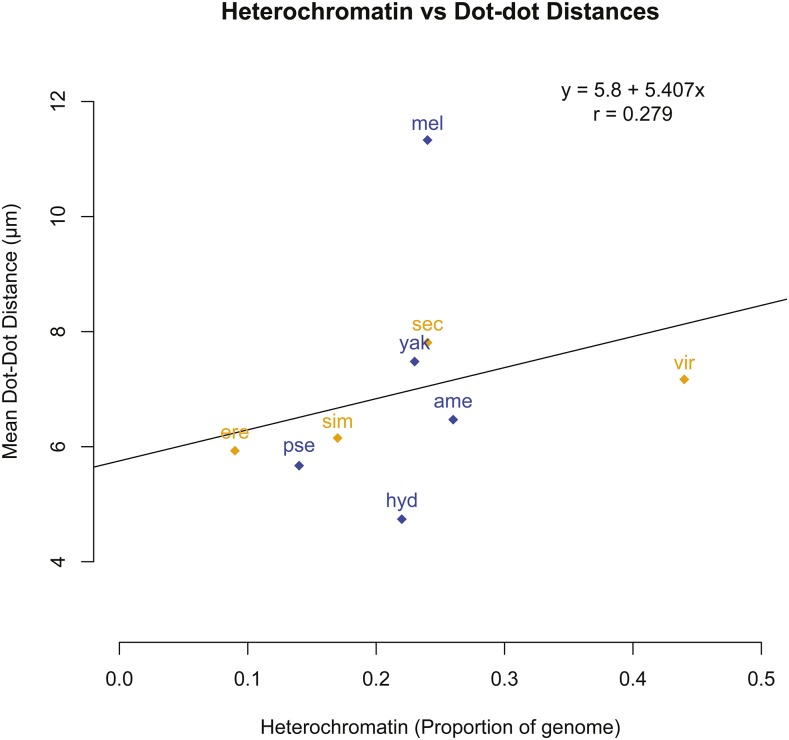
Dot-dot distances *vs.* genomic heterochromatin. For each species (see Table 1 for species abbreviations) the mean dot-dot separation distance is plotted against the proportion of the genome estimated to be heterochromatin. Blue and orange represent species with and without common inversions, respectively. While there was a positive correlation (r = 0.279) this was not statistically significant (regression analysis, *P* = 0.47).

We next asked whether the dot-dot distance was associated with the amount of heterochromatin in the genome, by plotting the proportion of heterochromatin against the mean dot-dot distance for the nine species for which we had heterochromatin estimates (Figure 3). This demonstrated that there was very little correlation between the amount of total genomic heterochromatin and the separation distance; for example, *D. virilis (vir)* has nearly 1.7x as much heterochromatin as its sister species, *D. americana (ame)*, but their dot-dot separation distances are very similar. If we ignore any effects of phylogeny, while there was a positive correlation between heterochromatin and distance (r = 0.279), this was not statistically significant (regression analysis, *P* = 0.47). These data fail to support the hypothesis that the total amount of genomic heterochromatin governs the separation distance between the dot chromosomes.

**Figure 4:**
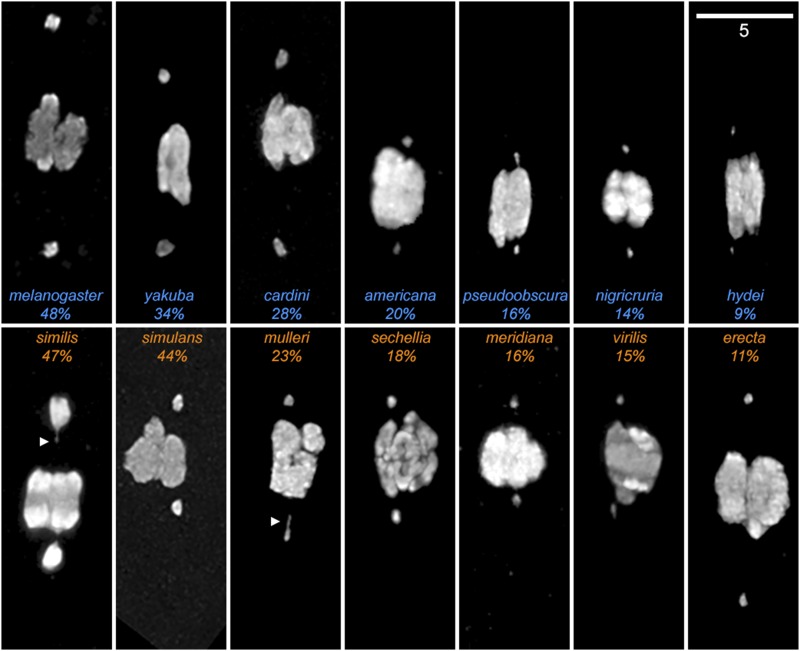
Karyosomes from the 14 *Drosophila* species showing the dot chromosomes out during Prometaphase I. DAPI staining of Prometaphase I chromosomes from each species with free dot chromosomes. Species are ordered by the proportion of Prometaphase oocytes with 1+ chromosomes out on the spindle (percentages). Each figure is rotated so the spindles are oriented vertically, with the species names colored by inversion type, with blue species having common inversions and orange species lacking common inversions. In addition to some chromosomes presenting visible chromatin threads coming from the dots (*e.g.*, *D. similis*, *D. mulleri*, arrowheads) there is considerable variation in the size of the dot chromosomes between species. (Scale bar: 5 µm).

**Figure 5:**
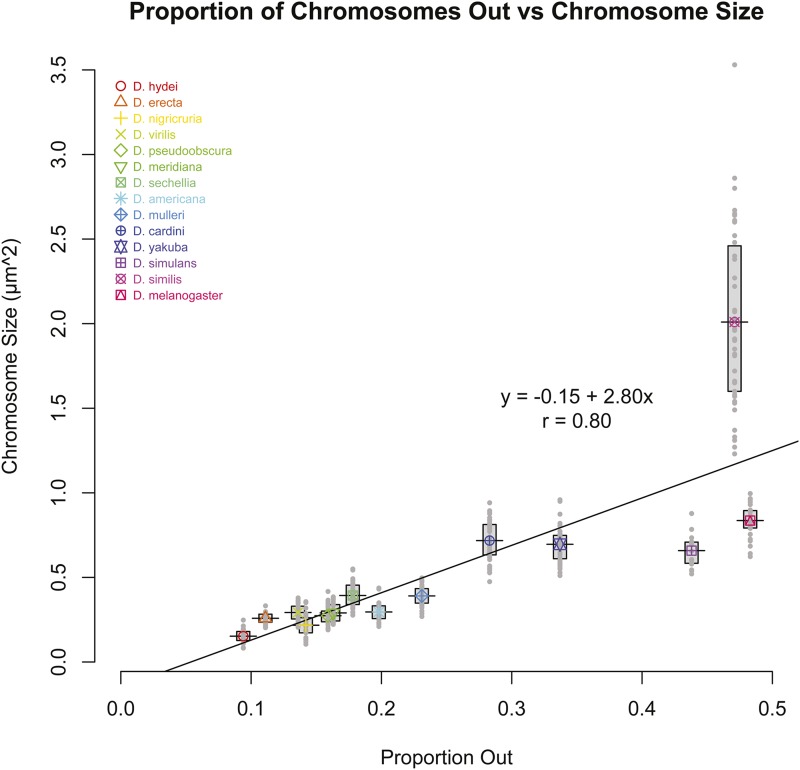
Proportion of oocytes with chromosomes out on the spindle *vs.* chromosome size. Each point is the cross-sectional area of a single measured chromosome, with the mean values (horizontal lines) and inner quartiles (gray boxes) indicated. Proportion out is calculated from data in Table 1. There is a strong correlation between the proportion of chromosomes out and the area of the chromosomes (r = 0.80). Note that *D. similis* is an extreme value, both in the mean chromosome size and in the amount of variation between oocytes; if that species is excluded, this correlation increases to r = 0.93.

In the course of collecting these data, we made two novel observations. First, we noticed that the dot chromosomes among these species appeared to vary greatly in size (Figure 4). This came as a surprise, but can be explained as many comparative studies of the F element have been conducted by examination of polytene salivary gland chromosome squashes (Leung *et al.* 2015) and heterochromatin is known to be underreplicated in polytene chromosomes (Leach *et al.* 2000). Therefore, the present study is the first time many of these species have ever had their chromosomes imaged in the normal cellular context of the Prometaphase spindle. Second, we noticed that in some species it was much easier to find oocytes that had chromosomes positioned out on the spindle than others; for some species we reached the target number of 30 oocytes in a single slide, while other species required combining multiple dissection preps to get sufficient material. To quantify these two observations, we measured the cross-sectional area of at least 20 dot chromosomes for each species, and calculated the proportion of all marked oocytes with one or more chromosomes out on the spindle. These measurements (Table 1) found that chromosome sizes varied among these species by more than 10-fold, while the proportion of chromosomes out varied by more than fivefold. Ignoring phylogenetic effects, we noted that while our measurements of dot-dot distance were well correlated with both chromosome size (r = 0.71), and with the proportion of chromosomes out (r = 0.74), the strongest pairwise correlation (r = 0.80) was between the proportion of chromosomes out and chromosome sizes (Figure 5). This correlation was also the most statistically significant (regression analysis, *P* < 0.001) of the three. This pattern is still retained if we correct for phylogenetic similarity by averaging the mean values of the species pairs used in Table 2 (while retaining *D. sechellia* and *D. pseudoobscura*), with the correlation between proportions of chromosomes out and dot chromosome sizes remaining at r = 0.80.

We note that in Figure 5 the dot chromosome of *D. similis* appears to be an extreme value, both in the size of the dot as well as in the amount of size variation among oocytes in this species. We considered the possibility that this species may no longer have a free dot chromosome, which would invalidate this comparison. However, while *D. similis* differs by two chromosome fusion events from *D. cardini*, its closest relative in our sample, neither fusion involved the *F* element (Cordeiro *et al.* 2014). Additionally, a larval brain squash appears to show the dot is simply a large and heavily heterochromatic chromosome in this species, with five other euchromatic chromosome arms evident (Figure S1). We therefore have not excluded *D. similis*, but note that if it were excluded the correlation between dot-dot distance and chromosome size across all species would increase to r = 0.77 while the correlation between the proportion of chromosomes out and chromosome size would rise to r = 0.93.

### Prometaphase without a free dot

**Figure 6:**
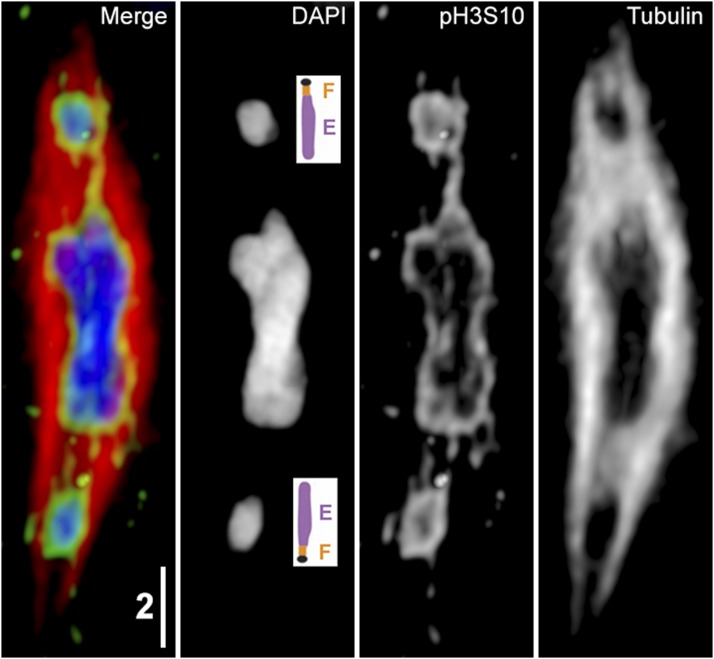
Nonexchange chromosomes have threads in *D. willistoni* Prometaphase I. A: A maximum intensity projection of a Prometaphase I *D. willistoni* oocyte, visualized with immunofluorescent staining of anti-phosphorylated histone-3 at serine 10 (pH3S10, yellow) and tubulin (red) with DAPI staining of DNA (blue), with both the merged figure and all three fluorophores shown individually. As the two chromosomes at the midzone are both metacentric, the nonexchange chromosome out on the spindle must be chromosome *3*, which is a telocentric chromosome with the Muller *E* element fused to the distal end of the *F* element (Schaeffer *et al.* 2008). As centromeres (DAPI panel inset, black dot) face the poles, the F element must be positioned closest to the spindle poles. While the chromatin threads connecting the nonexchange chromosomes are not discernible by DAPI staining, they can be seen with anti-pH3S10, as was originally observed in *D. melanogaster* (Hughes *et al.* 2011). Scale bar: 2 µm.

The preceding observations on the role of the dot chromosome led us to question what occurs during Prometaphase I in *D. willistoni*, a species that lacks a free dot chromosome. In this species, the Muller *F* element has fused with the *E* element (chromosome arm *3R* in *D. melanogaster)*, resulting in a karyotype consisting of two metacentric and one telocentric chromosomes, with the *F* element forming the centromere of telocentric chromosome *3* (Schaeffer *et al.* 2008). While this chromosome does recombine, rates of crossing over are still reduced within the *F* element, and this reduction spreads proximally into the *E* element (Powell *et al.* 2010). Examination of Prometaphase oocytes in *D. willistoni* females found that chromosomes could nevertheless still be found located out on the spindle during Prometaphase. While we did not have FISH probes to label the *F* element in this species, this element can be unambiguously identified, as it is positioned at the centromere of the only single-armed chromosome in the genome (Schaeffer *et al.* 2008). In all oocytes with cooriented chromosomes out on the spindle that we imaged, it appeared that the single-armed chromosome was the one that was out. As the centromeres face the spindle poles during Prometaphase (Hughes *et al.* 2009) this means that the *F* element must still be the part of the genome that is positioned closest to the spindle pole, even though it is no longer a free dot chromosome (Figure 6). To quantify the rate these chromosomes are out on the spindle, 15 mated females were dissected at 2 days post eclosion, and all oocytes were scored. Out of 115 oocytes that had passed Prophase, we found 14 (12%) with chromosomes out on the spindle. We also wanted to see if the heterochromatic threads connecting separated nonexchange chromosomes were also present in *D. willistoni*, so we performed immunofluorescent labeling of *D. willistoni* oocytes with antibodies against tubulin and pH3S10, a phosphospecific antibody that highlights the threads (Hughes *et al.* 2011). Like in *D. melanogaster* oocytes, we still saw threads in this species that lacks a free dot chromosome (Figure 6).

## Discussion and Conclusion

While our initial hypotheses were unsupported by the data, with neither the total amount of genomic heterochromatin nor the presence or absence of common inversions being significantly correlated with the dot-dot distances during meiotic Prometaphase, we did observe that there is substantial variation between species in both the size of the dot chromosomes and the proportion of oocytes with chromosomes out on the spindle. We further found that while the dot-dot distances were well correlated with both of these measures, the strongest correlation was between chromosome size and the proportion of oocytes with chromosomes out. To understand what the proportion of oocytes with chromosomes out implies, remember that these data were all collected from fixed images. Fixing a population of cells undergoing a dynamic process is analogous to recording multiple videos of someone repeatedly performing the same activity, then retaining only a single randomly-selected frame from the video of each repetition. This means that the more time cells spend in any particular configuration, the more frequently we expect to obtain fixed images of that configuration. Therefore, we can conclude that a species like *D. melanogaster*, with chromosomes out in 48% of oocytes, must be spending considerably more time undergoing the Prometaphase chromosome movements than a species like *D. hydei*, which has only 9% of oocytes with any chromosomes out.

One potential criticism of our test of the inversions hypothesis is that we did not attempt to assay whether the specific stocks of the tested species were currently heterozygous for inversions. The presence of inversions in the stocks could have resulted in greater distance measurements, as heterozygosity for an *X* chromosome balancer was found to increase the proportion of oocytes that had any chromosomes out on the spindle (Gilliland *et al.* 2009). This means that stocks from polymorphic species could have contained segregating inversions (it would be quite unexpected to isolate a segregating strain from a monomorphic population), which would have inflated the distances measured in the polymorphic species. However, even with this potential bias, the amount of variation within the polymorphic and monomorphic species groups was very similar (Figure 2), half of the species pair comparisons had greater distances in the monomorphic species, and the difference in mean distances between the groups was far from significant (*P* = 0.75). We therefore consider our results to be sufficient to reject the hypothesis that the ability for a species to tolerate common inversions has an effect on the separation of the dot chromosomes, although if a mutation could be found that changes whether a species tolerates inversions (like our working hypothesis predicts), it would be worth testing this again within one species. However, if that same mutation is also why balancer chromosomes work in *D. melanogaster* but not in *D. simulans*, then such a mutation is unlikely to ever be recovered. The phenotype of causing balancer failure must be one of the most obnoxious traits one could possibly study.

In hindsight, our initial focus on the total amount of genomic heterochromatin was naïve — the amount of heterochromatin on the dot chromosome would have been a more appropriate correlate. This can be seen in a species like *D. **vir**ilis*, which has the greatest amount of genomic heterochromatin of the analyzed species, yet also has a fairly small dot chromosome, indicating that we were wrong to assume that heterochromatin is equally distributed throughout the genome. However, the number of nucleotides on the dot chromosome is a hard number to obtain, given the difficulty of sequencing highly repetitive DNA, and highlights the need for more complete genome assemblies. Fortunately, recent advances in long read sequencing (Solares *et al.* 2018) should make assembling repetitive sequences considerably more tractable in the future, and give us better estimates of chromosome sizes. Nevertheless, if we use chromosome cross-sectional area as a proxy for the amount of DNA present, then the good correlation between dot-dot distances and chromosome size (r = 0.71) does provide significant support for the model that one function of heterochromatin is to construct the tethers connecting nonexchange chromosomes, and that the length of these tethers is limited by the amount of chromosome material available to build them. We should note there are both lower and upper limits on how far chromosomes can be separated, which likely attenuates the strength of this correlation. The minimum distance the dot chromosomes can be separated is the width of the exchange chromosomes at the spindle midzone; if the dots are closer together than that, then they are by definition not out on the spindle. Likewise, the maximum dot-dot separation is limited by the distance between the spindle poles. While the pole-pole distance increases and decreases over the normal course of Prometaphase (Gilliland *et al.* 2009) it seems reasonable that other factors besides dot chromosome size would affect maximum spindle length. With the previous observation that separation is reduced in flies heterozygous for *Df(4)m101-62f*, which deletes a visible amount of the pericentric heterochromatin (Gilliland, Colwell, Osiecki, *et al.* 2015), the present study provides additional evidence that heterochromatin content causes measurable changes in chromosome behavior.

The observation that chromosomes can still be found out on the spindle in *D. willistoni*, and that the *F* elements are still positioned closest to the spindle poles, is consistent with a model where the *F* element plays a role in organizing the meiotic spindle. While chromosome *3* is the only single-armed chromosome in the *D. willistoni* genome, and so the other metacentric autosomes may simply have never been spontaneously nonexchange in our relatively small number of figures, we note that Prometaphase chromosome movements were greatly reduced in *D. melanogaster* females with a compound-*4* and no homologous *4*, even when the *X* was forced to be nonexchange, and the compound-*4* was still closest to the pole despite lacking any pairing partner (Gilliland, Colwell, Lane, *et al.* 2015). Therefore, the present results are consistent with one of the roles of the dot chromosome being to enable chromosomes to move out on the spindle. This result also suggests an additional future direction, which is examination of meiotic Prometaphase in *D. ananassae*. While the *F* element in this species is still an independent chromosome, evolution has greatly expanded it into a much larger metacentric chromosome that is comparable in size to the other autosomes (Schaeffer *et al.* 2008). It would therefore be very interesting to see whether the *F* element still moves out on the Prometaphase spindle in *D. ananassae*, and if so, how the vastly increased amount of DNA on that chromosome affects the chromatin threads.
